# Bacteriophage Delivery Systems for Food Applications: Opportunities and Perspectives

**DOI:** 10.3390/v15061271

**Published:** 2023-05-29

**Authors:** Maria J. Costa, Lorenzo M. Pastrana, José A. Teixeira, Sanna M. Sillankorva, Miguel A. Cerqueira

**Affiliations:** 1 Centre of Biological Engineering, Campus de Gualtar, University of Minho, 4710-057 Braga, Portugal; 2 LABBELS—Associate Laboratory, Campus de Gualtar, University of Minho, 4710-057 Braga, Portugal; 3 International Iberian Nanotechnology Laboratory, Av. Mestre José Veiga, 4715-330 Braga, Portugal

**Keywords:** antibacterial, encapsulation, food contamination, foodborne diseases

## Abstract

Currently, one-third of all food produced worldwide is wasted or lost, and bacterial contamination is one of the main reasons. Moreover, foodborne diseases are a severe problem, causing more than 420,000 deaths and nearly 600 million illnesses yearly, demanding more attention to food safety. Thus, new solutions need to be explored to tackle these problems. A possible solution for bacterial contamination is using bacteriophages (phages), which are harmless to humans; these natural viruses can be used to prevent or reduce food contamination by foodborne pathogens. In this regard, several studies showed the effectiveness of phages against bacteria. However, when used in their free form, phages can lose infectivity, decreasing the application in foods. To overcome this problem, new delivery systems are being studied to incorporate phages and ensure prolonged activity and controlled release in food systems. This review focuses on the existent and new phage delivery systems applied in the food industry to promote food safety. Initially, an overview of phages, their main advantages, and challenges is presented, followed by the different delivery systems, focused in methodologies, and biomaterials that can be used. In the end, examples of phage applications in foods are disclosed and future perspectives are approached.

## 1. Introduction

In the last few years, the food industry has been growing at a fast pace, making food safety and quality assurance imperative. One of the major problems in the food chain is the presence of bacteria (e.g., spoilage and pathogenic) that cause enormous economic losses (e.g., food spoilage and consumer health issues) [1,2,3]. This major problem must be controlled and avoided to ensure food safety and quality and to guarantee the products shelf life. Food processing is one of the most important stages of the food chain, where several steps, such as unwanted temperature storage, cross-contamination, and unclean equipment, have been pointed to as causing undesired bacterial contamination, resulting in changes in the texture, flavour, colour and nutritional value of foods [4,5]. Regarding bacterial contamination, while spoilage bacteria cause food deterioration due to their metabolic activity, leading to the development of unpleasant properties in the odour, taste, and appearance of food [6], pathogenic bacteria have the potential to cause diseases when entering the body through different sources, such as food and water [7].

The microbiological safety criteria for food, namely the one pointed out by the Commission Regulation (EC) no. 2073/2005, is very strict and, in most cases, has zero-tolerance concerning the presence of the most common pathogenic bacteria related to foodborne diseases (i.e., *Listeria monocytogenes*, *Staphylococcus aureus*, *Escherichia coli*, *Campylobacter* spp., *Cryptosporidium* spp., and *Salmonella* spp.) [8,9]. Nevertheless, even with this regulation in place and with certified analytical reference methods, such as ISO 11290 and ISO 6579, which exist to assure food safety, every year, there are severe outbreaks linked to bacterial contaminants in foods that lead to countless foodborne diseases, causing millions of people to fall ill and thousands of deaths [10].

The most common examples of foods involved in outbreaks are eggs, poultry, raw milk, and other products of animal origin, mushrooms, fresh fruits, and vegetables. In 2021, the main outbreaks in Europe reported by the European Food Safety Authority (EFSA) were most frequent in composite foods and multi-ingredient foods and then in meat products. *Salmonella* was the most common pathogen involved in the outbreaks, and *L. monocytogenes* was the deadliest [11]. In 2022, in the USA, the occurred outbreaks were mainly related to *Salmonella*, *E. coli*, and *L. monocytogenes* that were present in a wide range of food products, from dairy to vegetables, fruits, composites, and frozen foods [12]. Foodborne illnesses in 2018 represented an estimated cost of 11.2 billion dollars in the UK and 17.6 billion dollars in the USA and in 2019, an estimated cost of 1.64 billion dollars in Australia [13,14,15].

To avoid and control food contamination, due to the presence of microorganisms, the food industry presents several strategies, such as: sampling to ensure the food is safe; food processing (e.g., freezing, cooking, preservatives) to control microbial growth; food storage using proper temperatures to avoid microbial growth; cleaning and sanitation of food contact surfaces to avoid cross-contamination and contamination spreading.

**Figure 1:**
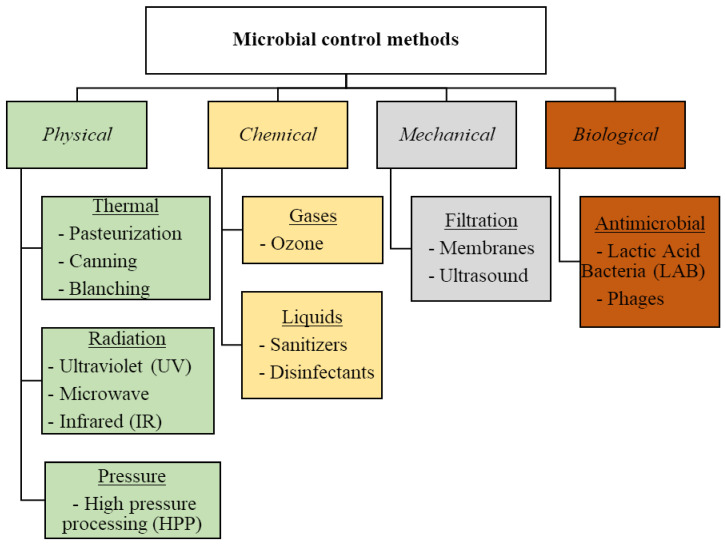
Examples of microbial control methods used in the food industry.

Microbial control methods are divided into physical (e.g., heat, dry), chemical (e.g., sanitizing agents), mechanical (e.g., filtering), and biological (e.g., microbial cultures), although biological control is less used in the food industry (Figure 1). For instance, chemical methods, such as disinfection, allow for the destruction or removal of pathogens (except endospores) or antisepsis using chemicals and applying them to surfaces. Physical methods, such as sterilization, achieve total microorganism removal; pasteurization, using mild heat below 100 °C, eliminates spoilage-causing microbes; irradiation, with sources, such as UV, reduces microorganisms; and high-pressure processing (HPP) inactivates foodborne pathogens. These methods enable the destruction or inhibition of pathogens and can be used in food processing, or after packaging [16,17,18]. However, the use of these traditional antimicrobial methods can negatively affect the food’s organoleptic properties and kill the beneficial bacteria that are present and needed in the food (e.g., cheese, yogurt) [19].

Among the biological methods, natural antimicrobials, such as phages, are valuable alternatives for decontamination due to phages’ high specificity for the targeted bacteria [20]. The use of phages as antimicrobials is drawing attention due to the growth of resistant bacteria in the last years, presenting a serious threat to public health, and phages have emerged as an efficient solution to help stop this crisis [21]. Another significant fact that increased the interest in natural antimicrobials is the actual consumers that turned their preference for natural, organic, and “clean label” foods, and are more concerned about the use of synthetic preservatives and demanding more natural solutions to preserve food [22,23]. There is a wide range of natural antimicrobials, from essential oils to bacteriocins, peptides, and phages, which can be used as an alternative to chemical antimicrobials [24,25,26].

Antimicrobials, such as phages, can be used in their free form; however, this approach presents some disadvantages, such as a fast loss of activity, non-controlled release, and different behaviour depending on the final application purpose (e.g., solid food, liquid food, food packaging). These issues are associated with the type of matrix used, the antimicrobials’ main characteristics, and the different interactions among the matrix, the antimicrobials, and foods [27,28]. Delivery systems are one of the strategies to overcome these issues through the use of bio-based structures, such as films, multilayer films, emulsions, particles, fibres and hydrogels that, according to the desired functionality (e.g., food formulation, food surface, food packaging), can be used to create innovative systems with antimicrobials for food applications [2,29,30].

This review presents the main considerations regarding phage advantages and challenges, namely the regulation. It also describes the types of delivery systems and biomaterials that can be used to incorporate phages and those with potential to be applied in the food industry. Finally, examples of delivery systems already applied to food products were gathered. To conclude, this review gives an outlook regarding the main knowledge acquired up to this moment and how it can be used for future developments.

## 2. Phages

### 2.1. History

Phages are the most abundant entities in the biosphere and are present in water and foods of diverse origins [31,32]. Phages meaning “bacteria eaters” in Greek, were discovered more than a century ago in 1896 by Ernest Hankin and in 1915 by Edward Twort, who reported an isolated filterable entity with the ability to destroy bacteria (antibacterial activity) and produce small clear areas on bacterial lawns (plaques). However, it was in 1917 that Felix d’Herelle reported the use of phages for therapeutical purposes to treat dysentery disease. From that moment until today, phage therapy has been successfully used in several bacterial diseases (e.g., typhoid, cholera) [33,34]. With the appearance of penicillin and other antibiotics, phage research started to be disregarded and marginalized in most of the world. However, some Eastern European countries and the former Soviet Union continued their phage research. In Georgia, phage therapy is part of general standard care used in paediatrics, burn treatment, and surgical hospital settings [32,33,35].

### 2.2. Properties and Applications

Phages are viruses that infect bacteria and have a narrow host range, usually strain-specific, meaning that each phage infects a specific bacterium. These highly specific entities are harmless for humans, animals, and plants [32,36]. Every day, humans are in contact with phages without any adverse effects. Phages are part of nature’s cycle, contributing to bacterial control in the environment (bacteria homeostasis) [37,38].

**Figure 2:**
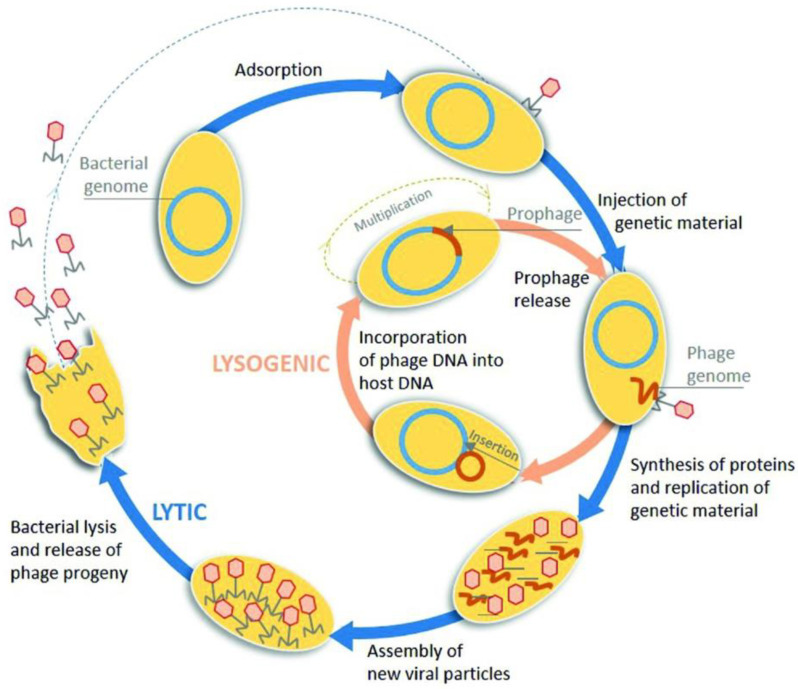
The lytic and lysogenic life cycles of phages. The blue arrows represent the steps to complete a lytic life cycle, and the orange arrows represent the lysogenic. Reprinted with permission from Pinto et al. [43], *FEMS Microbiol. Rev.*, Oxford University Press, 2021.

After binding to a specific bacterium, phages use the bacterial protein machinery to replicate inside and cause bacterial lysis and the release of newly formed viruses (lytic pathway), or after binding to the bacteria, phages integrate their genetic information into the bacterial chromosome without cell death (lysogenic pathway) (Figure 2). The most harmless approach is to use phages with a strict lytic pathway that causes bacterial death with no genome integration into the host DNA, in contrast to phages with a lysogenic pathway (that have the potential to create/transmit antibiotic resistance) [39]. 

Phages are classified and divided into different families depending on their morphology, nucleic acid composition, the structure of the capsid, and the host range. The International Committee on Taxonomy of Viruses (ICTV), more specifically, the Bacterial and Archaeal Viruses Subcommittee (BAVS), is responsible for phage taxa. Phages are divided into different morphotypes. *Caudiviricetes* comprises dsDNA tailed bacterial and archaeal phages. Non-tailed phages are distributed into different morphotypes, which include the *Corticoviridae* (circular dsDNA), *Tectiviridae* (linear dsDNA), *Plasmaviridae* (circular supercoiled dsDNA), *Cystoviridae* (tri-segmented dsRNA), *Leviviridae* (ssRNA), *Tubulavirales* (ssDNA phages), and *Microviridae* (ssDNA phages). The vast majority of phages identified to date belong to the *Caudoviricetes class*, which is currently divided into 7 orders, 44 families, and 44 subfamilies [40]. It contains phages having varying tail lengths with icosahedral capsids and a double-stranded DNA genome [41,42].These numbers, are however, in constant change due to the fast increase in available sequenced data.

Phages can be used in a wide range of applications concerning different areas, such as agriculture and aquaculture, acting as antimicrobials or for bacterial detection. In health, they can be used for the treatment of diseases and infection treatment and in microbiota modulation through phage therapy [44,45,46,47].

The use of phages has already been explored in some areas related to food, as a therapeutic agent, where phages are used to reduce pathogen colonization in living animals during primary production before slaughtering and also for sanitation purposes to disinfect food contact surfaces and equipment [48].

Phages can be used to prevent the contamination and proliferation of bacterial pathogens through storage of the final food products and for control purposes, where the bacterial colonization of food in industrial processing needs to be reduced [39]. They present several advantages as natural antimicrobials in the food industry [32,39,49] as follows:

- High specificity to their host;
- Self-replication (low dosages that will multiply as long as the host exists);
- Effectiveness in low doses;
- Safety;
- Easy and low-cost isolation and propagation (abundant in nature);
- Extension of food shelf life, avoiding bacterial contamination;
- Maintenance of food organoleptic properties (i.e., structure, taste, colour, odour) when using purified phages.

One of the main advantages of phages is related to their effectiveness in low dosages. This was shown, for instance, by Vikram et al. [50] using a cocktail of phages to reduce the levels of Shiga toxin-producing *E. coli* (STEC), namely *E. coli* O157:H7, in different types of meat, salmon, cheese, cantaloupe, and romaine lettuce. A significant STEC reduction (up to 97%) was accomplished in all foods when using 5 × 10^6^ PFU/g of phages against 10^3^ CFU/g of bacteria. Furthermore, the researchers also mimicked the real-life condition levels of STEC in food processing plants and on beef chuck roast samples, using phages (13 × 10^6^ PFU/g) to treat lower levels of *E. coli* O157:H7 (1 to 10 CFU/10 g). The prevalence of STEC was reduced by more than 80%, showing that even with a lower level of the bacterial host, these phages were able to self-replicate efficiently, which is another main advantage of phages.

Phages are very specific toward their specific hosts, which is of major importance in the food industry to prevent food contamination and ensure the maintenance of the food’s beneficial bacterial environment. Washizaki et al. [51] experimented using phage T4 against two different strains of *E. coli*—*E. coli* K12 and *E. coli* O157:H7—and found that the binding specificity was different due to the host’s structural difference. 

Besides food safety, organoleptic properties are very important and depend on the food quality. Perera et al. [52] tested the organoleptic properties of ready-to-eat (RTE) foods (e.g., lettuce, cheese, smoked salmon, and frozen entrées) after using ListShield against *L. monocytogenes*. In all the tested RTE foods, the organoleptic quality of the food remained stable with no differences observed in colour, taste, or appearance. In addition, ListShield caused the considerable reduction of *L. monocytogenes* (≥82%).

Due to phage effectiveness against specific bacterial strains when applied in food, phages can contribute to extending the food’s shelf life by decreasing or controlling bacterial contamination. Truchado et al. [53] used PhageGuard Listex and observed a reduction of 3.5 log in viable *L. monocytogenes* cells after 3 days of storage in fresh cut endive. Their work showed that phages are a promising method to decontaminate leafy greens, reducing bacterial growth and for shelf-life extension.

Phages are easy to isolate and propagate using a low-cost method. The vast majority of phages are fairly easily isolated from sewage and other sources. For instance, Sun et al. [54] isolated a bacteriophage (PSDA-2) against *Salmonella enterica serovar* Typhimurium from sewage and used it in chilled mutton. After 6 days, results showed a *Salmonella* reduction of 1.7 log in viable cells at 4 °C when a multiplicity of infection (MOI) of 100 was used and 2.1 log for a MOI of 10.000.

Phages are considered safe for human consumption without safety issues associated with oral ingestion [55]. In a review focused on the formulation, stabilization, and encapsulation of bacteriophages for phage therapy, Malik et al. [56] mentioned several studies that used phage suspensions in clinical trials showing no adverse effects on human health.

### 2.3. Consumer Perception and Legal Framework

In the current times, where the increasing trend is to consume more natural foods with fewer chemicals, phages have started to be noticed as possible and feasible solutions to use in foods as antimicrobials. Nowadays, the easy access to information regarding phages allows for a better understanding of the potential application of phages in food [57]. However, a long pathway needs to be implemented to improve the communication among science, the legal systems, the information media, and the consumers, to guarantee the information is received and processed clearly [58,59,60].

The use of phages in food applications in Europe is still very scarce due to legal restrictions. There is still a long way toward the approval of these novel solutions. The idea of using phages for bacterial control resurged twenty years ago due to the rapid increase in multi-drug resistant pathogens, but the phage-based products in the market today are still very few, mainly due to the lack of evolution in the legal framework. This European legal system is very restrictive, being a serious restraint to introducing phages, as antimicrobials, in the food industry. Moreover, since phages are isolated from nature, the intellectual property protection of phages is not possible, being an obstacle to companies that are interested in and able to invest in these types of solutions, together with the lack of stability over time [61]. In the last years, with the worldwide emergence of antibiotic-resistant bacteria, the regulatory status for phage application has changed, and some phage products have been approved for use in the food industry (Table 1). US Food and Drug Administration (FDA) recognized some phage-based products as Generally Recognised As Safe (GRAS). Phageguard Listex (previously called Listex) from Micreos Food Safety Company (Wageningen, The Netherlands) was approved in 2006, and in 2016, EFSA evaluated the safety and efficacy of Listex™ P100 for the reduction of pathogens on different RTE food products (EFSA Panel on Biological Hazards (BIOHAZ) & Hazards, 2016) positively. In addition, PhageGuard S. and PhageGuard E., from Micreos, were approved later for use against *Salmonella* and *E. coli* contamination in food. Omnilytics has FDA approval for a phage product against *E. coli* and *Salmonella* to treat live animals. FDA granted approval for LMP 102 from Intralytics Inc. from Baltimore, a phage cocktail against *L. monocytogenes*, in poultry packaging and ready-to-eat meat products. Following the United States, countries, such as Switzerland, Australia, New Zealand, Israel, and Canada, approved a few phage products to be applied to foods.

In Europe, EFSA is assessing the safety of phage-based products in food [62]. In 2019, the European Court of Justice issued an order that enables phages to prevent *Listeria* contamination in RTE foods despite the absence of a legal EU framework [63]. This decision can be seen as a clear message of the urgent need for an update on the legal restrictions that exist in the current days, regarding phage applications in the food industry.

**Table 1:** Phage-based products approved for food applications [19,31,50,64].

Manufacturer	Product	Application	Target Bacteria	Regulatory
Micreos Food Safety (Wageningen, The Netherlands) https://phageguard.com/ (accessed on 2 March 2023)	Phageguard Listex	Food	*L. monocytogenes*	FDA, GRAS Notice (GRN) 198/218; Food Standards Australia New Zealand (FSANZ); EFSA; Swiss BAG; Israel Ministry of Health; Health Canada
	Phageguard S	Food	*Salmonella*	FDA, GRN 468; FSANZ; Swiss BAG; Israel Ministry of Health; Health Canada
	PhageGuard E.	Food	*E. coli*	FDA, GRN 757
Intralytix Ltd. (Columbia, SC, USA) www.intralytix.com (accessed on 2 March 2023)	ListShield	Food	*L. monocytogenes*	FDA, 21 Code of Federal Regulations (CFR) 172.785; FDA, GRN 528; United States Environmental Protection Agency (EPA) Reg. No. 74234-1; Israel Ministry of Health; Health Canada
	SalmoFresh	Food	*Salmonella*	FDA, GRN 435; United States Department of Agriculture (USDA), Food Safety and Inspection Service (FSIS) Directive 7120.1; Israel Ministry of Health; Health Canada
	ShigaShield	Food	*Shigella* spp.	FDA, GRN 672
	Ecoshield PX	Food	*E. coli*	FDA, Food Contact Notification (FCN) 1018; Israel Ministry of Health; Health Canada
	SalmoLyse	Pet food	*Salmonella*	FDA, GRN 834, FCN 1018; Israel Ministry of Health; Health Canada
	ListPhage	Pet food	*L. monocytogenes*	-
	Ecolicide	Pet food	*E. coli* O157:H7	USDA, FSIS Directive 7120.1
Omnilytics Ltd. (Sandy, UT, USA) https://www.omnilytics.com/ (accessed on 2 March 2023)	Agriphage	Phyto	*Xanthomonas campestris* pv. *Vesicatoria*	EPA Reg. No. 67986-1
	Agriphage CMM	Phyto	*Clavibacter michiganensis* subsp. *Michiganensis*	EPA Reg. No. 67986-6
	Agriphage-Fire Blight	Phyto	*Erwinia amylovora*	EPA Reg. No. 67986-8
	Agriphage-citrus canker	Phyto	*X. citri* subsp. *Citri*	EPA Reg. No. 67986-9
	ECLYPSE-STEC	Food	*E. coli*	FDA, GRN 82
Phagelux (Nanjing, China) http://www.phageluxagrihealth.com/ (accessed on 2 March 2023)	Agriphage	Phyto	*X. campestris* pv. *vesicatoria, Pseudomonas syringae* pv. tomato	EPA Reg. No. 67986-1
	SalmoPro	Food	*Salmonella* spp.	FDA, GRN 752, GRN 603
Fixed phage (Glasgow, UK) https://www.fixed-phage.com/ (accessed on 2 March 2023)	safePHIX	Food	*E. coli*	-
	agriPHIX	Phyto	*Salmonella*	-
Passport Food safety solutions (West Des Moines, IA, USA) https://ahfoodchain.com/ (accessed on 2 March 2023)	Finalyse	Food	*E. coli* O157:H7	USDA, FSIS Directive 7120.1
APS Biocontrol (Dundee, UK) https://www.apsbiocontrol.com/products (accessed on 2 March 2023)	Biolyse	Phyto	*Erwinia*, *Pectobacterium*, *Pseudomonas*	-
Gum Products International’s (Newmarket, ON, Canada) https://www.gpiglobal.com/ (accessed on 2 March 2023)	GPI Biotech VAM-S	Food	*S. enterica*	FDA, GRN 917
Fink Tec GmbH (Hamm, Germany) https://www.finktec.com/solutions-for-industry/applied-phage (accessed on 2 March 2023)	Secure Shield E1	Food	*E. coli*	FDA, GRN 724

## 3. Phage Delivery Systems with Potential Use in Food Applications

Adding phages, in their free form, directly into food is possible. However, in most cases, this is not desirable due to faster activity loss, environmental processing conditions (e.g., pH, temperature), uncontrolled release, lack of bioavailability, and loss of stability during storage and along the passage through gastrointestinal conditions (i.e., raw food, vegetables, and fruits) [65,66]. Moreover, a specific range of MOIs is required to achieve the desired control and reduction of bacterial contamination, which is difficult to control when phages are used in the free form due to their uncontrolled release. To ensure that phages are delivered at a specific site and remain intact and viable, delivery systems can be used for their encapsulation [67]. 

Novel delivery systems using biomaterials where phages are incorporated (e.g., capsules, nanofibers, or emulsions) are being studied and are exhibiting promising results in the prevention and reduction of bacterial colonization, while allowing for prolonged activity of the phage and a potential controlled release [68,69,70]. The success of delivery systems will depend largely on the biomaterials used in their production and their specific characteristics (density, viscosity, solubility, surface tension). These biomaterials should be selected specifically according to the desired food application (e.g., food formulation, food surface, food packaging) and targeted food products (e.g., fruits, vegetables, meat).

The selection of the biomaterials to produce phage delivery systems must consider the properties of the incorporated phage to ensure phage compatibility and activity maintenance. Polysaccharides, proteins, and lipids are the most used biomaterials for delivery system production with intended use in food applications [71]. For phages, water-soluble materials, such as polysaccharides and proteins from natural sources, are ideal since phages have better maintenance of their stability and viability when stored in water-based solutions (e.g., buffers) [56,72,73].

Delivery systems can be produced using different techniques that allow for a diverse range of applications in food (e.g., food processing, food packaging, food contact surface) depending on the final purpose (e.g., sanitation, preservation, control, modulation) and on the active substance that this system will deliver (e.g., phages, probiotics, essential oils). There are several techniques, such as spray drying, freeze drying, emulsification, extrusion, or electrospinning, which are studied and can be explored as delivery systems. 

These strategies allow for the production of structures with different scales (nano to macro) and can allow for effective retention and the possibility of controlled release of the phages at different rates and in specific sites [74,75]. 

At the same time, while phage viability is improved, the interaction with other elements that are part of the same environment (i.e., food matrix) is reduced, being less susceptible to environmental factors (e.g., pH and temperature, promoting phage stability) [76]. Moreover, when used for surface treatment or in packaging, the type of delivery system produced and the type of application in the food (e.g., spraying, dipping, immersion, wrapping) should consider the manufacturer’s equipment, requirements, and the final application. Bio-based structures, such as films, multilayer films, emulsions, capsules, and hydrogels, according to the desired functionality, can be used to create innovative phage delivery systems for food applications.

### 3.1. Extrusion

The most common methodology for encapsulation is extrusion, a simple, low-cost methodology that enables viability of the loaded compound over time and can be applied using the conventional dropwise method or with a nozzle. Extrusion is performed by dropping the biopolymer solution into a crosslinker solution or by dropping the crosslinker into the biopolymer solution [77,78]. 

One of the most used materials to produce particles or beads using extrusion is sodium alginate. Men et al. [29] used alginate microcapsules to protect phage J25 that targets *S. aureus* because when in its free form, it becomes inactive after 6 h at 20 °C. It was possible to achieve an encapsulation efficiency of 87.43%, and the J25 phage stability was significantly improved with minimal titre loss. After 35 days of storage at 4 °C and 20 °C, the phage titre was 10^9^ PFU/mg (0 log loss) and ≈10^7^ PFU/mg (≈2 log loss), respectively. When tested in liquid food, the alginate microcapsules also showed that the number of *S. aureus* was significantly reduced, and that these capsules can be used to prevent contamination in fresh milk, egg white, and broth when used at 0.1, 1 and 2 g/kg, respectively. 

Besides using different materials, these can also be used together in an extrusion method to incorporate phages. Samtlebe et al. [75] tested three different methods to incorporate phages, the emulsion method with enzymatically gelled milk protein, extrusion with alginate crosslinked with calcium chloride (CaCl_2_), and extrusion with alginate and whey protein isolate (WPI) crosslinked with calcium chloride (CaCl_2_). P008 phages were encapsulated, and it was observed that the addition of whey protein to alginate and further crosslinking with CaCl_2_ led to a phage titre of 10^7^ PFU/g after 120 min in simulated gastric fluid at pH 2.5, while for crosslinked alginate without WPI obtained through extrusion, the phage titre after 30 min was less than 10^1^ PFU/g. This difference was explained based on the whey protein’s interference with the diffusion of simulated gastric fluid (SGF) components into the capsule due to the buffering capacity of milk proteins and due to the possible formation of a hydrophobic surface and a crosslinked protein network. This results from the interaction between the positive charge of whey protein when the pH is lower than the isoelectric point (pI ≈ 5.2), which will interact with the negatively charged alginate molecules, conferring more resistance to an acid environment [79].

Some biomaterials with antimicrobial properties can be used to carry phages or cocktail phages and promote a synergistic antimicrobial effect against different bacterial strains. Rahimzadeh et al. [80] used chitosan nanoparticles to encapsulate a bacteriophage cocktail and target *S. enterica*, *Shigella flexneri*, and *E. coli* in rats as a treatment for bacterial diarrhoea. The treatment effect in the weight loss and in the presence of positive cultured stools was observed. The rats administered the chitosan-encapsulated bacteriophage cocktail maintained their weight after 3 days and had lower group weight changes, while in rats with Cefixime administered, there was significant weight loss. In rats infected with *S. enterica* and *S. flexneri*, positive cultured stools were reduced after 2 days and null after 6 days with the chitosan-encapsulated bacteriophage cocktail. When using Cefixime, 4 days were necessary for a reduction, and after 8 days, there were 50% positive culture stools. These results show that phage cocktails can have a synergistic effect against pathogens and show the promising potential of using this type of system as a treatment for gastrointestinal infections with better results compared to traditional treatment. This type of system can also be used in food products to avoid contamination by these bacteria since their materials are safe and these bacteria are also responsible for the main outbreaks in foods.

### 3.2. Emulsification

Emulsification is another process where “a stable dispersion of two or more immiscible liquids held in suspension by small percentages of substances called emulsifiers” [81]. This technique allows for the production of smaller particles, but the high stirring rate necessary for its production can influence the antimicrobial activity (i.e., phage or probiotic). 

Different types of emulsions can be used to incorporate phages: water-in-oil (W/O), oil-in-water (O/W), water-in-oil-in-water (W/O/W) complex emulsions [82]. Balcão et al. [68] created a water-in-oil-in-water (W/O/W) emulsion to entrap phages (nanovesicles with ca. 85–200 nm average size). Phages were entrapped in the aqueous core of the nano balloons and the system showed a long storage time (3 months) and stability (zeta potential ca. −12 mV). Regarding the phages, their antimicrobial activity was accessed in vitro against *S.* Enteritidis, and it could be observed that a low antibacterial activity was obtained using 5 mg of lyophilized phage, but when using 10 mg, significant antibacterial activity was observed (no quantitative results were presented).

Active delivery systems that respond to a specific environmental condition, such as pH or temperature, can also be created using emulsions. Vinner et al. [83] used microencapsulation to create a pH-responsive solid oral dosage formulation containing enteric phages using a scalable membrane emulsification process. The study used Eudragit^®^ S100 (not approved for food applications), alginate, polyglycerol polyricinoleate (PGPR), Miglyol, and castor oil to produce the microparticle (W/O) emulsion using a batch membrane emulsification dispersion cell. Phage viability remained stable in refrigerated conditions over 4 weeks. Significant phage protection was observed after prolonged exposure to the simulated gastric acidic environment, and this system was also shown to be effective in killing *E. coli* in in vitro cell cultures and human epithelial cells.

Besides using different types of emulsions and select biomaterials that allow for the production of an active delivery system, the oils used in the emulsions can be selected according to their antimicrobial properties promoting a synergistic effect with the phage antimicrobial properties. Moon et al. [84] combined the beneficial effects of essential oils with phages and used emulsions as a delivery system for the control of *Salmonella* in chicken meat. A commercial bacteriophage solution was used (Salmonelex), and 10 essential oils were tested. The most active essential oils against *Salmonella* were thymol and carvacrol. The emulsions were produced by dissolving the essential oils in propylene glycol and Tween 80 using a mechanical homogenizer. The results showed that when applied sequentially, Salmonellex and Thymol and Salmonellex and carvacrol presented better results than when used alone. One drawback was the emulsion stability due to phase separation. Nanoemulsions can be used to improve stability. Although high stirring rates can present an issue to phage stability, which should be considered when using nanoemulsions. 

### 3.3. Spray Drying

Spray drying is another possible strategy that allows for a high production rate and is an easy and reproducible methodology. However, the high temperatures used during the drying process can result in a problem when incorporating temperature-sensitive compounds. In spray drying, a liquid mixture (solution, emulsion, or suspension) is atomized in a hot gas to obtain a powder in the drying chamber [85]. In spray drying, the drying temperature, spray mesh size (large, medium, size), spray cap size, and feed rates are all controlled parameters that will have a direct influence on the produced particles. Using this technique, particles with different morphologies and sizes (smooth spherical particles to shrivelled-wrinkled, mixed-wrinkled, doughnut-shaped, and granules) are produced. Spray drying can also be used to produce dry powders carrying bioactive agents, which are easier to handle and with long storage stability at ambient temperature [86]. When using spray drying to encapsulate phages, the temperature, humidity, and the matrix glass transition properties are the main factors to ensure phage viability.

Spray drying was already used in some studies to create phage delivery systems for human ingestion. Vinner et al. [85] microencapsulated the Felix O1 phage specific for *Salmonella* bacteria using spray drying to apply as a solid oral dosage form for humans. The Eudragit S100^®^ pH-responsive copolymer (not approved for food applications) was used together with trehalose (used to protect Felix O1 phage from thermal stresses and acidic conditions). The protection of Felix O1, when exposed to SGF, was effective, with titre maintenance (10^9^ PFU/mL) when using 2% Eudragit in the formulation and 1% trehalose. Forming tablets through direct compression was also shown to significantly improve phage protection under acidic conditions. Trehalose was able to protect Felix O1 from temperature conditions used in spray drying for different temperatures (100 °C, 120 °C, 150 °C, 180 °C) and Eudragit was able to protect the phage from the acidic conditions when exposed to SGF (pH 2) for 2 h. When trehalose was used alone to protect Felix O1, the phage had poor acid stability (pH 2), and trehalose at higher concentrations reduced the phage stability. Eudragit alone resulted in a lower Felix O1 titre after spray drying (5 log loss) due to a lack of thermal protection. The formulation with 1% (*w*/*v*) trehalose with 2% Eudragit (*w*/*v*) was selected based on temperature and acid resistance and its pH-responsive behaviour showed promising potential to be used for phage oral delivery, to target *Salmonella* in the gastrointestinal tract. 

Besides these studies focused on the gastrointestinal tract, other research has also focused on human applications targeting the lungs. Chang et al. [87] produced a highly stable phage formulation to treat *Pseudomonas aeruginosa* lung infection using spray drying. Formulations with trehalose, lactose, or leucine showed less than a 1 log reduction in the phage titre during spray drying, where lactose showed better results regarding phage protection compared to trehalose. Sugar and leucine were found to be determinants to maintain phage viability and stability, since without sugar and leucine, there was a 6.7 log to full reduction in the phage titre.

In other studies, besides different polymers, other materials like sugar and sugar alcohols (e.g., mannitol) and amino acids (e.g., leucine and glycine), were used to potentiate phage viability and stability [88]. Materials, such as trehalose ,were also used in another study, and it has been shown that when using trehalose as an excipient for the phages in spray drying, the increase in relative humidity caused the crystallization of the matrix leading to phage destruction [89]. 

### 3.4. Electrospinning

Electrospinning is also a technique of encapsulation that is gaining interest by the research community for the production of delivery systems due to its several advantages: different sizes range (micro to nano), possibility of different structures (fibres and particles), no temperature or organic solvent requirement, ability to scale up, and a versatile morphology [90]. 

Different types of biomaterials can be used to incorporate phages and can be used in electrospinning. Vonasek et al. [91] immobilized bacteriophage T7 on electrospun cellulose microfibers using different approaches: non-specific adsorption, protein–ligand binding, and electrostatic interactions. Electrostatic interactions displayed the best results, with a phage loading efficiency between 15 and 25% and indicated slow release over a period of 24 h showing the potential to immobilize phages on biomaterial surfaces. 

Different approaches can be performed to improve phage viability using materials, such as gelatine, which together with the biopolymers and with the optimized conditions, allow for phage viability and the production of nanofibers. Costa et al. [92] used polyvinyl alcohol (PVOH) to encapsulate the Felix O1 phage. SM buffer with 0.01% gelatine was used as a solvent to improve phage stability, and optimized conditions of 0.3 mL/h and 25 kV allowed for the production of homogeneous nanofibers and presented a high activity of phage Felix O1 with a small log loss (2 log PFU/mL) during the production process, showing the promising application of these nanofibers, to be used in food packaging, to prevent and control *Salmonella* contamination in foods. 

Similar to extrusion and emulsion delivery systems, in electrospinning, synergies can be created by blending biopolymers with antimicrobial materials that can potentiate the antimicrobial effect of the phage delivery system. A recent research study used *Aloe vera* to incorporate *Pseudomonas* sp. Phages in nanofibers through electrospinning. The results showed a change in the morphology when adding the phages to *Aloe vera* and thermal stability and flexibility properties were improved, and there was a synergistic effect when using *Aloe vera* and phages regarding the antimicrobial properties, revealing the potential to be used as a hybrid nanomaterial for food packaging [93].

### 3.5. Coatings and Films

Active coatings and films can control and prevent foodborne pathogens when used directly in foods or used for the functionalization of packaging by incorporating antimicrobial compounds, such as phages, that have a specific target, depending on the type of food where the packaging will be applied. Coatings present advantages suitable to be used in packaging or even to be applied directly to food. They serve as a barrier to moisture, lipids, and gases, guaranteeing food quality and when used as carriers for antimicrobials. Food safety is also improved, avoiding microbial contamination and extending food’s shelf life [94].

Huang & Nitin [65] produced a WPI-based coating loaded with phages and applied them on fish feed to treat bacterial infections caused by *E. coli* and *Vibrio* spp. in the aquaculture industry. The results showed enhanced stability (≤1.6 log PFU/pellet after a one-hour exposure to simulated gastric conditions) compared with the controls where, after 30 min, no phages were detected. A bacterial reduction of 3 and up to 5 log in the simulated intestinal digestion was observed, showing their potential application to treat fish infections.

Another type of whey protein, whey protein concentrate (WPC), was used by Kamali et al. [95] with pullulan (PUL) to produce composite films and incorporate Bacteriophage A551. An anti-*Listeria* effect was observed during storage after 60 days at 25 °C. Three different ratios were tested, 70 WPC:30 PUL, 50WPC:50 PUL, and 30 WPC: 70 PUL. The composite film with the best performance was 30 WPC:70 PUL regarding mechanical and physical properties and phage incorporation efficiency and stability. Regarding the phage stability, there was a 3-log PFU/cm^2^ reduction after 60 days (initial titre 10^7^ CFU/cm^2^, final titre 10^4^ CFU/cm^2^). Regarding antibacterial properties, the composite film with the best performance was 70 WPC:30 PUL in the growth inhibition assay (liquid medium) with an approximately 4 log CFU/cm^2^ reduction after 24 h. However, regarding the inhibition in solid medium, 30 WPC:70 PUL displayed the best results since it was the only one that still showed inhibition after 60 days. These different results are probably related to the type of lysis. The authors report that phages lysis from without occurs in solid medium, promoting these differences. This is an interesting result for food applications since depending on the type of food (liquid or solid), the results can differ.

To ensure the efficiency of the delivery systems in food applications, different matrices should be studied and compared for encapsulated phages. This will help to uncover the range of available options and will allow an improvement of these systems. Dicastillo et al. [73] used three different matrices to encapsulate ListexP100 using sodium caseinate, sodium alginate, and polyvinyl alcohol as biomaterials. It was observed that the morphology, colour, opacity, and thermal stability were maintained with phage incorporation. Regarding the antimicrobial effect, it was possible to observe that when used in free form, the phage (10^8^ PFU/mL) significantly reduced the bacterial load, although for higher concentrations of bacteria (10^6^ CFU/mL), the inhibitory phage effect was reduced (1.2 log after 3 h), probably related to the low MOI used, since an MOI of 10,000 was completely effective (no bacterial growth). Regarding the phage incorporated into films, a reduction in *L. monocytogenes* was achieved when measured at 1, 3, and 24 h, and the PVOH films presented the highest reduction (≈1.6 log CFU/mL). Regarding the PVOH films, these performed better in refrigerated conditions, reaching a 2 log CFU/mL reduction after 8 days of incubation. An interesting fact was that an increase in the bacterial load was observed for the first two days, associated with the slow release of the phages from the PVOH matrix, and also due to the temperature since the phage’s optimal temperature was 30 °C and these results were obtained in refrigerated conditions. In this system, the free phage had better results regarding antimicrobial properties, probably related to the pH of the delivery systems and the casting method that, due to drying conditions, can promote stress and desiccation. Nevertheless, this system showed potential to be used as an active coating or film in food against *L. monocytogenes*.

Table 2 summarizes information regarding phage delivery system materials with the potential to be used in the food industry and in microbiological modulation.

## 4. Phage Delivery Systems Used in Food Products

There are a few studies that have explored phage delivery systems using biopolymers and evaluated their effectiveness in food products [2,24,91,102]. Table 3 shows works regarding delivery systems intended for food applications. 

Most of the studies with applications in food products used different types of meat to test the delivery systems, since these are a main concerns regarding foodborne diseases. 

### 4.1. Emulsification

**Figure 3:**
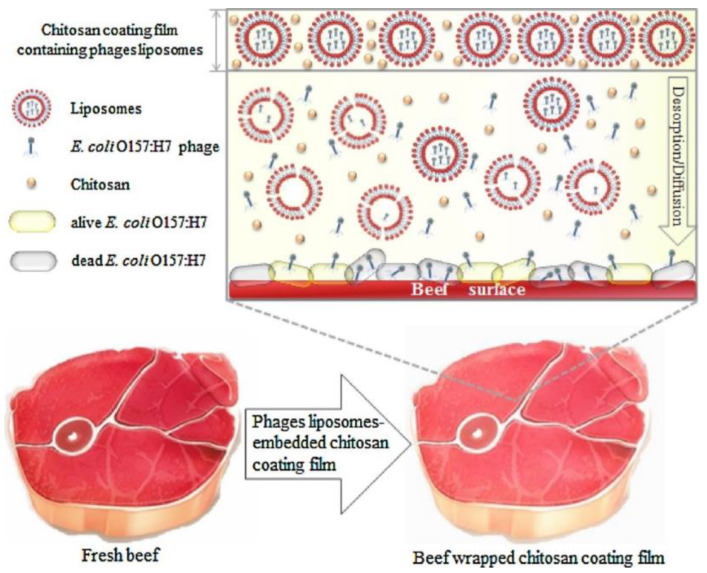
Antibacterial process of chitosan film containing liposome-encapsulated phages against *E. coli* O157:H7. Reprinted with permission from Cui et al. [101], Carbohydrate Polymers, *Elsevier*, 2017.

Cui et al. [103] explored a chitosan film’s potential with a liposome-encapsulated phage to control *E. coli* O157:H7 in beef (Figure 3). The phage encapsulation efficiency was around 57%, and high antibacterial activity against *E. coli* O157:H7 was observed. The sensory properties of the beef were tested and remained the same after 7 days. The phage was isolated and incorporated into a chitosan 1% solution with liposome particles formed with soy lecithin and cholesterol (5:1 *w*/*w*) and dissolved in chloroform. The phage suspension was added to 2% PBS solution. A reduction of around 5 log CFU/g in *E. coli* (control had 7.8 log CFU/g) was observed after 6 days of beef storage at room temperature conditions. This system shows potential to be applied in meat food applications due to organoleptic and antimicrobial properties. 

Another study used phages coated with poly-L-lysine liposomes for application to pork to treat *E. coli* O157:H7, and their effect was studied by Lin et al. [104]. Poly-L-lysine (PLL) was used to enhance the stability of the phages and was produced with soy lecithin, cholesterol, and poly-vinylpyrrolidone. An encapsulation efficiency of 57.59% was achieved, and a reduction of 2.44 log CFU/mL of *E. coli* O157:H7 was achieved after one day of incubation at pH 5.5 in pork suspension using 10^9^ PFU/mL of phage and a 40% volume ratio of PLL liposomes, extending the treatment time, without significant impact on the quality of the pork products, regarding colour and a sensory evaluation (appearance and smell). After 7 days, liposomes had better sensory evaluation results at three different temperatures (4 °C, 12 °C, and 25 °C) compared with the control. The temperature showed no influence on the coated phage’s antibacterial activity. Phages applied in free form could slowly reduce the number of *E. coli* O157:H7 cells in the first three days, but after that, an increase in *E. coli* was observed after 6 days. On the other hand, the PLL liposomes with phages were able to control *E. coli* proliferation after 15 days at 4 °C.

### 4.2. Coatings and Films

Alves et al. [2] used sodium alginate-based films crosslinked with calcium chloride to load phage ϕIBB-PF7A and analyse their preventative effects on *Pseudomonas fluorescens* in poultry. The films demonstrated a decrease in phage viability only after 8 weeks in refrigerated conditions. The antimicrobial efficacy was analysed in artificially inoculated chicken breasts, and a decrease of 2 log CFU/mL in the first 2 days was achieved, maintaining a reduction until day 5. The use of the film was found to be a good approach for use in the food industry to prevent microbial spoilage. Subsequent research with a phage cocktail (EC4 and φ135) was used with cinnamaldehyde based on sodium alginate emulsion-based films against *Salmonella* Enteritidis and *E. coli* [96]. 

The results showed that cinnamaldehyde did not compromise phage incorporation and that the phage incorporation maintained the sodium alginate film parameters. The combination of cinnamaldehyde with EC4 and φ135 promoted a synergistic effect against *E. coli* and *S.* Enteritidis, respectively. In this study, 0.4% of cinnamaldehyde (CNMA) promoted a reduction of 5.7 log CFU/mL based on *E. coli*. When combined with φ135, CNMA promoted a reduction of 7 log CFU/mL of *Salmonella* on the chicken surfaces, and for EC4, the same reduction (7 log CFU/mL) in *E. coli* was observed, revealing the potential of this approach for use in food packaging to prevent food contamination.

In an interesting strategy, Gouvêa et al. [105] used absorbent pads usually used in chilled meat trays and applied a cocktail of six isolated phages (FSE16, BFSE18, PaDTA1, PaDTA9, PaDTA10, and PaDTA11) and evaluated the reduction in the initial count of *S.* Typhimurium present in the environment. The highest reduction of 4.36 log CFU/mL was achieved for a 10^9^ PFU/mL concentration and an 80% (*v*/*w*) solution applied in the pads at 15 °C. At 10 °C, the phage effect was less efficient due to the slower bacterial metabolism. The phages remained viable in the pads for 48 h. If in this study, the phages were loaded into a coating, such as in the work of Alves et al. [2], and then applied to the absorbent pads, the viability of the phages would probably be greater, and at the same time, their effect over time on *S.* Typhimurium would increase and could be applied in food packaging (e.g., meat packaging). 

Radford et al. [106] evaluated the antimicrobial properties of Felix O1 and *Listeria* phage A511 when embedded in a xanthan gum solution that was used to coat poly(lactic acid) films. The active films were then used in the packaging of precooked sliced turkey breast. It was realized that the growth of *Salmonella* and *L. monocytogenes* was inhibited in precooked sliced turkey breast after 30 days in anaerobic packaging at 4 °C or 10 °C. Reductions of 0.832 log CFU/cm^2^ at 4 °C and 1.30 log at 10 °C for *Salmonella* sp. and 6.31 log CFU/cm^2^ at 4 °C and 1.52 log CFU/cm^2^ at 10 °C for *L. monocytogenes* were observed. The *Listeria* phage A511 coating also inhibited the growth of *L. monocytogenes* over 14 days in aerobic packaging (3.79 log CFU/cm^2^ at 4 °C, 2.17 log CFU/cm^2^ at 10 °C). These results showed the potential of using phage-loaded coatings to functionalize packaging for the control of *L. monocytogenes* contamination in refrigerated foods. 

Another study evaluated the effect of polycaprolactone (PCL) films with phage T4 against *E. coli* O157:H7 in raw beef and analysed the antibacterial activity for 120 h at 10 °C. The films were chemically functionalized films using alkaline hydrolysis followed by an 1-ethyl-3-[3-dimethylaminopropyl]carbodiimide hydrochloride EDC/NHS activation followed by a crosslinking with phage T4 and presented a significantly higher reduction than physically adsorbed films. The physically adsorbed films were produced by dipping the PCL film into the phage T4 suspension. In the in vitro system, these results were more expressive, probably related to a lower diffusion rate and the difference in the water content between LB medium solution and raw beef [107]. The fact that PCL-chemically functionalized films had a homogenous distribution while the physically adsorbed one presented a heterogenous distribution, probably due to surface properties, can also contribute to a lower efficacy. Another study that could be performed would be the distribution of the bacteria in the meat to understand these differences between in vitro and the raw beef results.

Besides meat, delivery systems with loaded phages also showed promising results in fruits, such as tomato. Amarillas et al. [30] used chitosan-based edible coatings loaded with phage to fight *E. coli* O157:H7 on tomato surface sand observed that the lytic activity of the phage (vB_EcoMH2W) was maintained without a significant loss. For one week, the bacterial growth was reduced by 3 log after 6 days at 20 °C, when compared with the control, which presented an *E. coli* O157:H7 concentration of ≈10^6^ CFU/g when using a phage concentration of 10^6^ PFU/mL in the chitosan coatings. The tomatoes were submerged in the chitosan solution for 1 min, the excess was drained, and afterwards, the tomatoes were inoculated by immersion with an *E. coli* solution of 10^7^ CFU/mL for 10 min, dried, and stored.

**Table 3:** Phage delivery systems used in food products.

Phage	Main Microorganism	Material(s)	Delivery System	Food Product Application	Phage Titre in Delivery System	Control Bacterial Log	Bacterial Log Variation *	Ref.
φIBB-PF7A	*P. fluorescens*	Alginate	Film	Chicken fillets	10^5^ PFU/cm^2^	10^6^ CFU/cm^2^	≈0.5 log reduction after 2 days ≈3 log increase after 7 days	[2]
JS25	*S. aureus*	Alginate	Microcapsule	Fresh milk Liquid egg white Broth	10^9^ PFU/mg (2 g/kg)	10^3^ CFU/g	After 12 days at 4 °C and 20 °C Fresh milk—3 log (complete reduction) Liquid egg white—3 log (complete reduction) Broth—3 log (complete reduction)	[29]
Felix O1	*S.* Typhimurium	Xanthan on polylactic acid (PLA)	Coating on film	Precooked sliced turkey	10^9^ PFU/mL	10^3^ CFU/cm^2^	*S.* Typhimurium 0.832 log reduction at 4 °C 1.3 log reduction at 10 °C *L. monocytogenes* 6.31 log reduction at 4 °C 1.52 log reduction at 10 °C	[106]
A511	*L. monocytogenes*	Xanthan gum on PLA	Coating on film	Precooked sliced turkey	10^9^ PFU/mL	10^6^–10^7^ CFU/cm^2^	6.79 log reduction at 4 °C 2.17 log reduction at 10 °C	
*S.* Enteritidis F5–4 *S.* Typhimurium L2–1 *S.* Typhimurium ICB1–1	*S.* Enteritidis *S.* Typhimurium	WPC Carboxymethyl cellulose (CMC) Chitosan Alginate	Coating	Strawberries	10^8^ PFU/mL **	10^5^ CFU/g	After 5 days at 4 °C WPC—3.1 log reduction CMC—1.4 log reduction Chitosan—2.0 log reduction Sodium alginate—1.9 log reduction	[108]
vB_EcoMH2W	*E. coli* O157:H7	Chitosan	Coating	Tomato	10^6^ PFU/mL	10^6^ CFU/g	3 log reduction	[30]
T4	*E. coli* O157:H7 H17130	PCL	Film	Beef	10^9^ PFU/mL (Value of the solution used)	10^5^ CFU/mL	After 5 days at 10 °C Chemically functionalized film—1 log reduction Physically adsorbed film—≈ no reduction	[107]
phiIPLA-RODI stock	*S. aureus* IPLA1	Gelatine	Coatings Films	Cheese	1.75 × 10^8^ PFU/mL (GF1) 1.16 × 10^8^ PFU/mL (GF2) 6.35 × 10^7^ PFU/mL (GF3)	10^6^ CFU/mL	After 6 days at 4 °C GF1 coating—≈1.5 log reduction GF2 coating—≈1.5 log reduction GF3 coating—≈1.5 log reduction GF1 film—2 log reduction GF2 film—≈0.5 log reduction GF3 film—≈0.5 log reduction	[109]
T7	*E. coli* BL21	WPI, beeswax	Coating	Cucumbers Apple Tomatoes	10^7^ PFU/mL	Sliced cucumbers 10^5^ CFU/sample Cut apple 10^8^ CFU/sample Tomato 10^5^ CFU/sample	Sliced cucumbers—0 log—no reduction Cut apple—2 log reduction Tomato—≈1.5 log reduction	[110]
*Myoviridae* DT1 to DT6	*E. coli* DH5α *E. coli* EPEC *E. coli* non-O157 STEC *E. coli* O157 STEC	WPC	Film	Meat	5.2 × 10^7^ PFU/film	*E. coli* DH5α ≈ 10^2^ CFU/mL *E. coli* EPEC 10^3^ CFU/mL *E. coli* non-O157 STEC 10^2^ CFU/mL *E. coli* O157 STEC 10^2^ CFU/mL	After 24 h at 4 °C *E. coli* DH5α—2 log reduction *E. coli* EPEC—≈no reduction *E. coli* non-O157 STEC—≈no reduction *E. coli* O157 STEC—2 log reduction After 24 h at 24 °C *E. coli* DH5α—increase ≈ 1.5 log *E. coli* EPEC—increase ≈ 5.5 log *E. coli* non-O157 STEC—increase ≈ 4.5 log *E. coli* O157 STEC increase ≈ 4 log	[111]
*E. coli* isolated phage	*E. coli* O157:H7	PLL	Liposome	Pork	0.8 × 10^10^ PFU/mL	≈10^3^ CFU/g	After 15 days at 4 °C—≈0.5 log reduction After 15 days at 12 °C—≈0.5 log reduction After 15 days at 25 °C—≈0.1 log reduction	[104]

* Bacterial log variation compared to the initial control log value. ** Phage titre loaded into the coating films.

## 5. Conclusions and Future Perspectives

Phages can be used as valuable natural antimicrobials in the food industry. There is a wide range of applications from the pre- and postharvest stage, to serve as sanitizers and to apply to food products, always with the purpose of protecting the food against bacterial contamination.

Future research should focus on the creation and optimization of delivery systems chosen specifically based on the exposure temperature, pH (food and delivery system), and type of food product (e.g., solid or liquid) that is going to be used and the type of pathogen and specific strains that can be a source of contamination in the selected food products. Studies performed until now showed that applications using antimicrobial materials, such as chitosan, can have a synergistic effect when combined with phages, increasing the antimicrobial activity. Moreover, the use of essential oils and other antimicrobials, such as *Aloe vera*, showed a synergistic effect, fostering the creation of novel systems with these combinations. Furthermore, the use of bacteriophage cocktails in the reported studies showed a synergistic effect against pathogens, something that can be accessed to minimize bacterial levels. This is a good option, especially when dealing with different bacteria and even different strains, since phages have different antibacterial effects depending on the strain used. MOI is of major importance, since lower levels can lead to ineffective delivery systems and higher levels promote antibacterial activity.

Regarding the materials used, the milk proteins showed a good capacity for phage protection in some delivery systems due to their buffering capacity and potential binding to the matrix, something to take into consideration when producing novel delivery systems. Sugars, such as lactose and trehalose, can be used to increase phage viability due to protection regarding different temperatures and shear stress. The functionalization of delivery systems, such as films, can be an interesting approach since it allows for better phage dispersion on the delivery system and a higher antimicrobial effect.

Phage long-term stability and a satisfactory phage incorporation efficiency are very important, and their evaluation should be tested. Moreover, bacterial control and inspection in the processing environment must be evaluated to perceive possible issues of bacterial resistance to phages. These are all major considerations to have, when creating phage delivery systems, before they become accepted and used in the food industry, to ensure the successful outcome of these systems.

The legal framework needs an urgent update, especially in Europe, to allow for phage applications in the food industry, ensuring that all previous issues listed above are analysed and reported. With the change in the legal framework panorama, further work will be necessary to adapt phage production and the novel delivery systems to large-scale production, following good manufacturing practices.

An improvement in communication with the companies and consumers must be achieved to ensure full acceptance and improve their interest in these applications, which will promote research on this topic, legal framework consolidation, consumer understanding, and the consequent acceptance of food products using these applications.

If these considerations are taken into account, a faster increase in the research regarding phage delivery systems for food applications is foreseen. The consequent application of these solutions in the market will help food industry to prevent foodborne diseases, deaths, and food waste.

## Figures and Tables

**Table 2:** Phage delivery systems to be used in the food industry.

**Phage**	Polymer(s)	Delivery System(s)	Main Microorganism(s)	Conditions	Phage Initial Titre	Phage Final Titre	Ref.
EC5 and φ135	Alginate	Films produced by casting microcapsules produced by emulsification	*E. coli* *S.* Enteritidis	**Sample**—2 × 2 cm^2^ film **Solution**—2 mL SM buffer **Time**—45 min **Temperature**—Room temperature (RT)	EC4—10^9^ PFU/cm^2^ φ135—10^9^ PFU/cm^2^	10^6^ PFU/cm^2^—EC4 10^6^–10^7^ PFU/cm^2^—φ135	[96]
UFV-AREG1	Alginate	Microcapsules produced by extrusion	*E. coli* O157:H7	**Sample**—Microcapsule suspension **Solution**—Broken-microsphere solution (MBS) **Time**—Few minutes (until dissolution) **Temperature**—RT *	10^6^ PFU/mL	10^5^ PFU/mL	[97]
T4	Polyethylene oxide(PEO)/cellulose diacetate	Nanofiber produced by electrospinning	*E. coli*	**Sample**—1 g nanofiber **Solution**—10 mL SM buffer **Shaking**—120 rpm **Temperature**—RT * Time—10 min	10^8^ PFU/mL	10^7^–10^8^ PFU/mL	[98]
T4	Alginate	Dry macrospheres produced by extrusion	*E. coli* DSM-613	**Sample**—1 g phage-loaded beads **Solution**—99 mL MBS **Time**—180 min **Temperature**—37 °C	≈10^10^ PFU/mL	>10^9^ PFU/mL	[99]
T7	Cellulose acetate Chitosan Poly(ethyleneimine) (PEI)	Microfibers produced by electrospinning	*E. coli* BL21	**Sample**—1 cm microfiber disk **Solution**—2 mL water **Time**—24 h **Temperature**—RT	10^5^–10^6^ PFU/mL	10^5^–10^6^ PFU/mL	[91]
K1F	Eudragit S100/Alginate	Microcapsules produced by emulsification	*E. coli* EV36-RFP	**Sample**—1 g phage loaded beads **Solution**—99 mL MBS **Time**—180 min **Temperature**—37 °C	≈10^9^ PFU/mL	≈10^9^ PFU/g	[83]
T7 Vibrio phage (#11985-B1)	WPI	Coating	*E. coli* BL21 *Vibrio* spp. (#11985)	**Sample**—1 coated fish pellet **Solution**—1 mL maximum recovery diluent (MRD) **Time**—2 min + 1 min vortex **Temperature**—RT *	10^7^ PFU/mL	≈10^5^ PFU/pellet	[65]
A551	WPC/Pullulan	Composite films produced by casting	*L. ivanovii* *L. monocytogenes*	**Sample**—15 mm diameter disk film **Solution**—1 mL water **Time**—5 min **Temperature**—25 °C	10^9^ PFU/mL	10^5^ PFU/cm^2^—70WPC:30PULL 10^6^ PFU/cm^2^—50WPC:50PULL 10^7^ PFU/cm^2^—30WPC:70PULL	[95]
Listex P100	Alginate/Gelatine Sodium caseinate PVOH	Films produced by casting	*L. monocytogenes*	**Sample**—15 mm diameter disk film **Solution**—1 mL water **Time**—5 min	10^9^ PFU/15 g of film forming solution	N/A	[73]
JS25	Alginate	Microcapsule produced by extrusion	*Staphylococcus aureus*	**Sample**—5 g Freeze dried microcapsules **Pre-hydration**—15 mL SM Buffer, 37 °C, 24 h **Solution**—45 mL microsphere-broken solution (MB) **Time**—1 h **Temperature**—20 °C	10^12^ PFU/g	10^9^ PFU/mg	[29]
P008	Alginate, WPI	Capsules produced by emulsification Capsules produced by extrusion	*L. lactis* subsp. *lactis* biovar. *diacetylactis* F7/2	**Sample**—1 g capsules **Solution**—9 mL 50 mM sodium citrate, 200 mM sodium hydrogen carbonate and 50 mM Tris-HCl (pH 7.5) **Time**—15 min **Temperature**—37 °C	10^12^ PFU/mL	10^9^ PFU/g	[75]
Felix O1	Polyhydroxybutyrate/polyhydroxyvaleratePHBV/PVOH	Films produced by casting Nanofibers produced by electrospinning	*S.* Enteritidis	**Sample**—25 cm^2^ film or nanofiber **Solution**—10 mL SM buffer **Time**—1 h **Shaking**—120 rpm **Temperature**—25 °C	10^8^ PFU/mL 10^7^ PFU/mL	10^6^ PFU/mL 10^6^ PFU/mL	[92]
Phage Team1	Alginate	Micro-beads produced by extrusion	*S. aureus*	**Sample**—Beads **Solution**—20 g/L trisodium citrate **Shaking**—Stomacher 1 min **Time**—15 min **Temperature**—25 °C	10^9^ PFU/mL	10^9^ PFU/mL—Fresh 10^9^ PFU/mL—Frozen 10^4^ PFU/mL—Lyophilised	[100]
phiIPLA-RODI	Lipo-NTM Nio-NTM Phospholipon 90G	Nanovesicles produced by emulsification	*S. aureus*	**Sample**—Centrifuged 1.5 mL of niosomes/transfersomes/liposomes **Solution**—PBS (washing) 30 µL chloroform, SM Buffer up to 1.5 mL **Temperature**—4 °C **Time**—15 min (final centrifugation) **Centrifugation**—10,000 rpm	10^8^ PFU/mL	0.5 to 1 log loss—10^8^ PFU/mL Liposome 1.2 log loss—10^7^ PFU/mL Niosome 1.3 log loss—10^7^ PFU/mL Transfersome	[101]
phiIPLA-RODI	Alginate	Microcapsules produced by extrusion	*S. aureus*	**Sample**—1 g microcapsules **Solution**—9 mL, 0.1 M sodium citrate **Temperature**—RT * Time—20 min	10^9^ PFU/mL	10^6^ PFU/mL	[70]
Phage cocktail	Chitosan	Nanoparticles produced by extrusion	*S. enterica* *Shigella flexneri* *E. coli*	Entrapment efficiency **Centrifugation**—19.980 g, 30 min **Filtration**—pore size 0.22 µm	10^10^ PFU/mL	10^9^ PFU/mL	[80]
phi-2/2	Softisan/LutrolF68	Nanovesicles produced by emulsification	*S.* Enteritidis	**Sample**—1000 µL emulsion nanovesicles **Solution**—20 µL chloroform **Temperature**—RT * Time—5 s vortex + 10 min centrifugation	10^9^ PFU/mL	Low activity	[68]

* RT was assumed when no temperature was mentioned.

## Data Availability

Not applicable.
